# Experimental and Numerical Investigation of the Effect of Different Shapes of Collars on the Reduction of Scour around a Single Bridge Pier

**DOI:** 10.1371/journal.pone.0098592

**Published:** 2014-06-11

**Authors:** Afshin Jahangirzadeh, Hossein Basser, Shatirah Akib, Hojat Karami, Sareh Naji, Shahaboddin Shamshirband

**Affiliations:** 1 Department of Civil Engineering, University of Malaya, Kuala Lumpur, Malaysia; 2 Department of Civil Engineering, Semnan University, Semnan, Iran; 3 Department of Computer Science, Chalous Branch, Islamic Azad University (IAU), Chalous, Mazandaran, Iran; Centro de Investigacion Cientifica y Educacion Superior de Ensenada, Mexico

## Abstract

The scour phenomenon around bridge piers causes great quantities of damages annually all over the world. Collars are considered as one of the substantial methods for reducing the depth and volume of scour around bridge piers. In this study, the experimental and numerical methods are used to investigate two different shapes of collars, i.e, rectangular and circular, in terms of reducing scour around a single bridge pier. The experiments were conducted in hydraulic laboratory at university of Malaya. The scour around the bridge pier and collars was simulated numerically using a three-dimensional, CFD model namely SSIIM 2.0, to verify the application of the model. The results indicated that although, both types of collars provides a considerable decrease in the depth of the scour, the rectangular collar, decreases scour depth around the pier by 79 percent, and has better performance compared to the circular collar. Furthermore, it was observed that using collars under the stream’s bed, resulted in the most reduction in the scour depth around the pier. The results also show the SSIIM 2.0 model could simulate the scour phenomenon around a single bridge pier and collars with sufficient accuracy. Using the experimental and numerical results, two new equations were developed to predict the scour depth around a bridge pier exposed to circular and rectangular collars.

## Introduction

The collapse of bridges after a flood is a disaster resulting in irrevocable consequences such as interrupting transport systems, injuring and taking lives. It has been claimed that over 1000 bridges have been failed in the last 30 years in the United States and 60% of those failures were associated with scour at the base of the bridge [1]. Bridge scour is known for its negative impact on highway bridges in the United States [2]. The level of this danger was emphasized by the results of a study conducted by the Transport Research Board, which indicated, at that time, there were approximately 488,750 bridges spanning streams and rivers in the United States and that $30 million were spent each year to deal with scour-associated bridge collapses [3].

**Figure 1:**
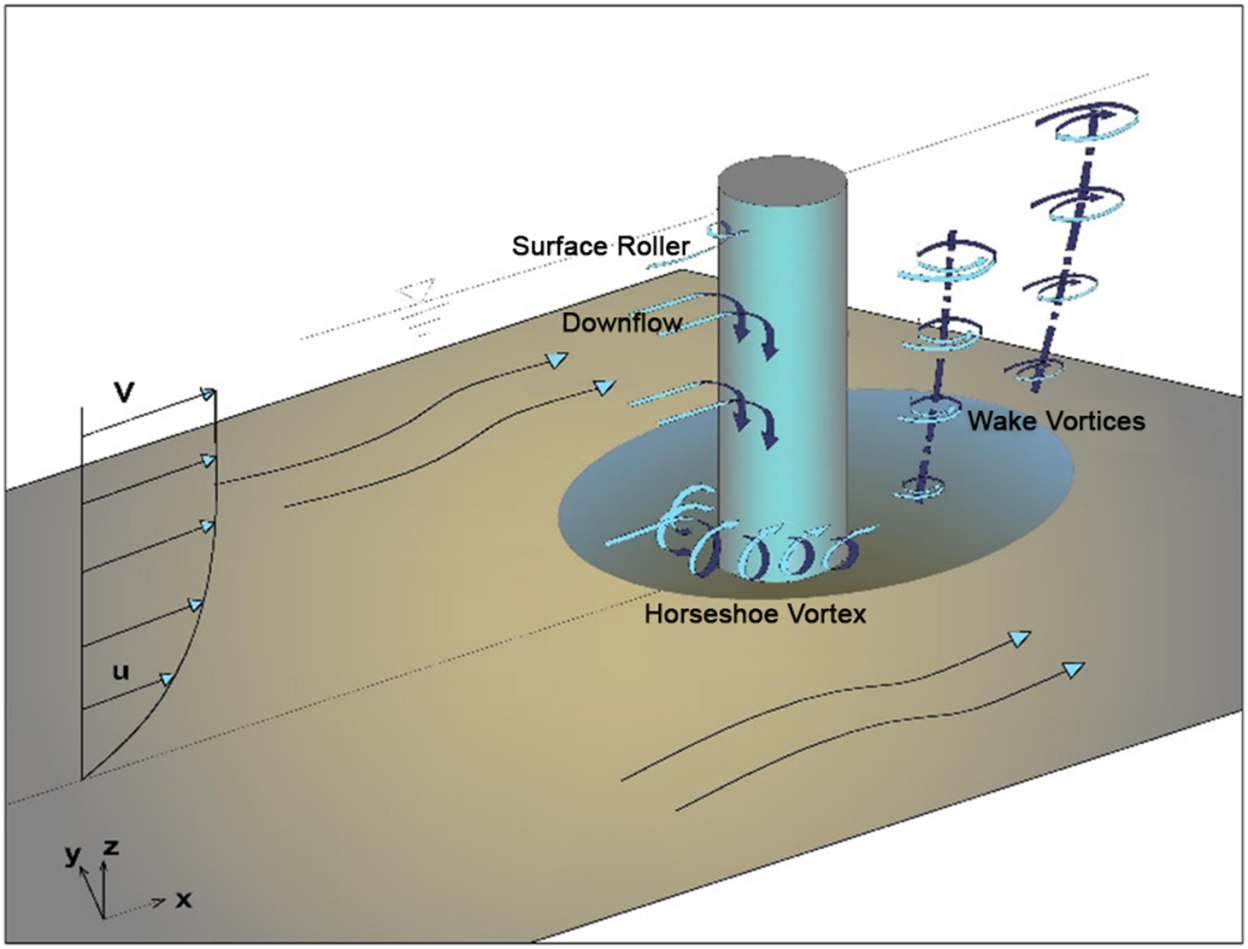
Flow and scour pattern around a cylindrical pier.

The key factor in the development of a scour hole is the presence of complex vortex systems around the bridge piers (Figure 1). When the flow impacts the nose of the pier, the flow causes an adverse pressure gradient in the upstream side of the pier, resulting in three dimensional boundary layer separation in front of the pier. Then a downward flow is developed due to the downward negative stagnation pressure gradient of the logarithmic approach flow adjacent to the upstream face of the pier. This downward flow and its interaction with boundary layer separation close to the bed forms a vortex system [4]. This vortex system which has a shape similar to a horseshoe, is called the horseshoe vortex and known as the main local scouring mechanism at the base of the bridge piers [5]. Moreover the separation of the flow downstream from the pier forms wake vortices, which function as little ‘tornados,’ lift material from the streambed and generating an independent scour hole at downstream side of the pier [6].

In order to protect bridge piers against scouring, different methods and countermeasures were used by researchers. These methods are grouped generally into two distinct categories, i.e., armoring and flow-altering methods, which also are known as direct and indirect methods, respectively [7]. In the armoring method, the structures are protected directly against scouring using various approaches, such as riprap stones, reno-mattresses, cabled-tied blocks, gabions, tetra pods, dolos, concrete-filled mats and bags, and concrete aprons. In the flow-altering methods, the flow pattern is modified by using sacrificial piles, sills, collars, slots, and other methods to reduce scour.

Numerous researchers have addressed the scour phenomenon and developed different types of countermeasures for local scour at bridge piers. These researchers include Raudkivi and Ettema, 1983; Odgaard and Wang, 1987; Breusers and Raudkivi, 1991; Bertoldi and Kilgore, 1993; Parola, 1993; Melville and Raudkivi, 1997; Parker et al., 1998; Melville and Chiew, 1999; Melville and Hadfield, 1999; Zarrati et al., 2006; Link et al., 2008; Christensen, 2009; Ghodsian and vaghefi, 2009; Akib et al., 2011; Akib et al., 2014 a,b; Jahangirzadeh et al. 2014; Termini, 2011; and Kumar et al., 2012 [8]–[26]. Also the scour-reduction efficiency of collars was established in earlier studies, including Schneible, 1951; Thomas, 1967; Tanaka and Yano, 1967; Neill, 1973; Chiew, 1992; Dey, 1997; Kumar et al., 1999; Zarrati et al., 2004; Alabi, 2006; SaniKhani et al., 2008; Moncada et al., 2009 and Euler and Herget, 2012 [27]–[38].

Researchers have used different shapes of collars around cylindrical and rectangular piers to control the scour. However, a comprehensive study to compare the effect of the shape and geometry of collars on scour around a single bridge pier has not been done. For example Kumar et al., (1999) used a series of circular collars with different dimensions only at the bed level to control scour around a cylindrical bridge pier [33]. Zarrati et al., 2004 examined the effect of collar elevation on the scour reduction around a rectangular pier just by using one shape of collar [34]. In this respect, this study investigates the application of circular and rectangular collars around cylindrical bridge piers with different elevations above and under the streambed using both experimental and numerical methods. In addition to the experimental studies mentioned above, a few CFD models have been developed for computing flow patterns and bed-profile changes around hydraulic structures, e.g., SSIIM, Fluent, and Flow-3D models. The simulation of sediment transport processes around bridge piers requires at least a two-dimensional, hydrodynamic and sediment-transport model [39]. In the present study, SSIIM 2.0, a three-dimensional model, is used to compute the transport of sediment around a single bridge pier, and an evaluation of its capability for simulating the scour around hydraulic structures is carried out.

The results of this study can be used by researchers and engineers as the basis for designing and performing future research projects in this area. Novel equations, numerical parameters, and sediment formulas are studied, and some recommendations are provided in order to solve scour problems.

## Materials and Methods

**Figure 2:**
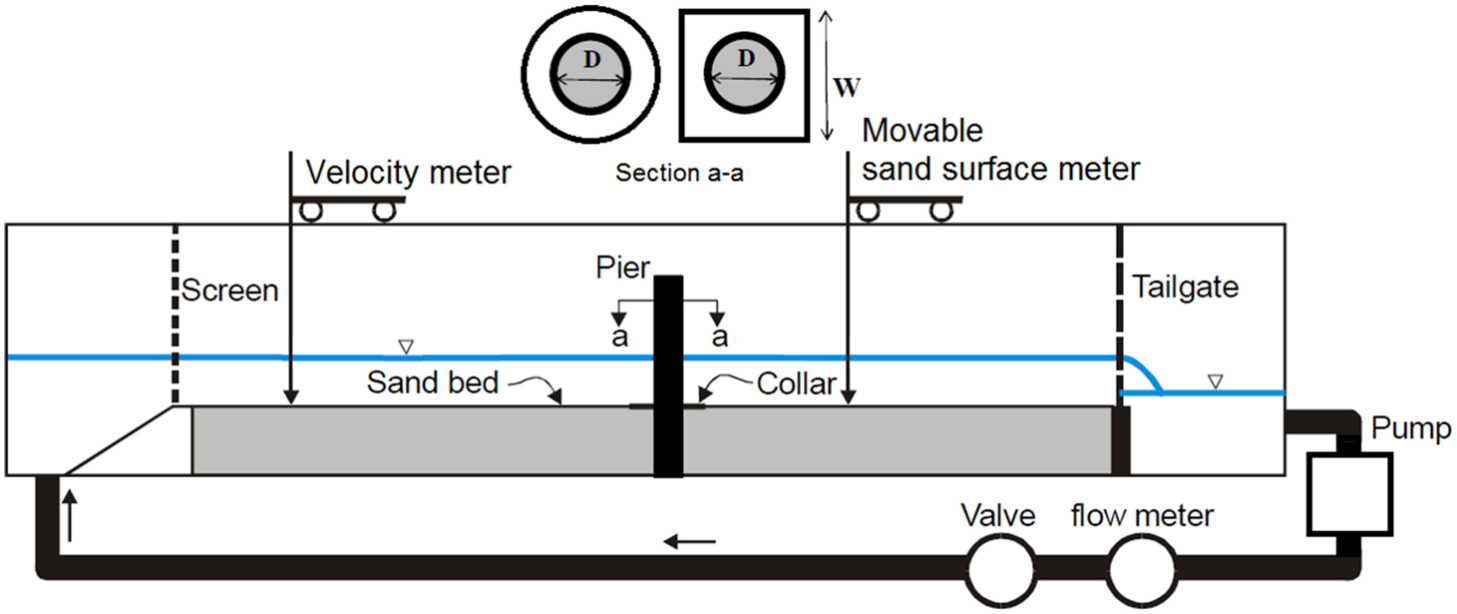
Schematic view of the experimental flume and single bridge pier.

The experiments in this study are conducted in the Hydraulic Laboratory at the University of Malaya. A rectangular section of a flume with 12 m length, 30 cm width, and 45 cm depth is used in the experiments. The bed and sides of the flume are made of glass supported by a metal frame. Figure 2 shows the schematic view of the flume and the experimental bridge pier. To ensure the existence of a uniform flow and a fully-developed turbulent flow upstream of the test sections. The velocity profiles were measured during all tests using a 3-axis electronic current velocity meter. The rate of the discharge was measured using a sharp-edge weir calibrated and embedded at the entry gate. Two pumps were used to circulate the water, and an adjustable tail gate was used to measure the depth of the water. The flow velocity and depth of scouring are measured and recorded using a 3-axis electronic current velocity meter and a Sand Surface Meter. The experimental model scaled from a river conditions during floods and with usual dimensions (width = 20 m, water level = 2.4). The dimensions of flume and water level was simulated based on considering scale factor of 1/20. For simulating water velocity and sediment size, Froude criterion and Shields diagram is used respectively. The sediment in prototype is assumed to be fine gravel.

The flume was filled with uniform bed sediment (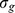
<1.4) to a thickness of 0.15 m. The sediment has a median size (*d_50_*) of 0.8 mm, a specific gravity (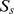) of 2.65, and a geometric standard deviation (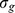) of 1.37. To avoid wall effects on the rate of scour, the maximum diameter of the pier was set to 10% of the width of the flume (3 cm) based on Chiew and Melville’s (1987) recommendations. Also the following condition was satisfied to eliminate the effect of the sediment on the depth of the scour:


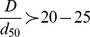
 [15]. According to the two above-mentioned conditions, the pier diameter is set to 3 cm.

To develop the experimental model of a single pier a cylindrical pier made of plexiglass with a diameter of 3 cm was used. The depth (Y) of the flow in the approach always was set to 12 cm. This value is selected based on the recommendation of Chiew and Melville (1987), i.e., flow depth (Y) >3.5×diameter (D) of the pier, to eliminate the pier’s effect on the rate of scour [40].

The experiments were conducted with a flow intensity of *U/U_cr_* = 0.95 (flow intensity = the ratio of the velocity of the approaching flow to the critical velocity for incipient motion for bed sediment movement, i.e., *U/U_cr_*). The critical velocity for incipient motion for bed sediment movement (*U_cr_*_)_ is 0.36 m/s based on the shields diagram. Thus the velocity of the approaching flow is set to 0.34 in all experiments.

The clear-water condition, i.e., *U/U_cr_*<1, was satisfied in all experiments to reach the maximum scour depth. Clear-water condition is where the flow velocity is too low for general sediment transport to occur.

In order to investigate the effects of the shape of the collar on the scour depth, two different shapers of collars are used. In this respect, rectangular and circular in four different sizes are chosen to be investigated in this study, i.e., 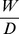 = 2.0, 2.5, 3.0, and 3.5 where D is the diameter of the pier, and W is the width of the rectangular collar or the diameter of the circular collar. In this study, plates with a thickness of 0.8 mm were used for making the physical models of collars. Three heights of 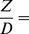
−0.5, 0.0, and 0.5. Z is the distance of the collar from the surface of the bed, were used to investigate the influence of the collar level on the depth of the scour. The maximum test duration for the experiment was 72 hours. The criterion used to ensure the achievement of equilibrium condition in which, the change in the depth of the scour was 5% or less than pier diameter over a period of 24 hours [15], [41]. The depth of the scour did not change more than 5% of the pier diameter over a period of 24 hours. Therefore, the equilibrium state was reached in 72 hours.

At the end of each experiment, the water was drained from the flume with sufficient care to prevent any disturbance in the scour holes. Then the variations of the bed profile around the pier were measured by a sand surface meter.

## Results and Discussion

### 1. Experiments without Collars

**Figure 3:**
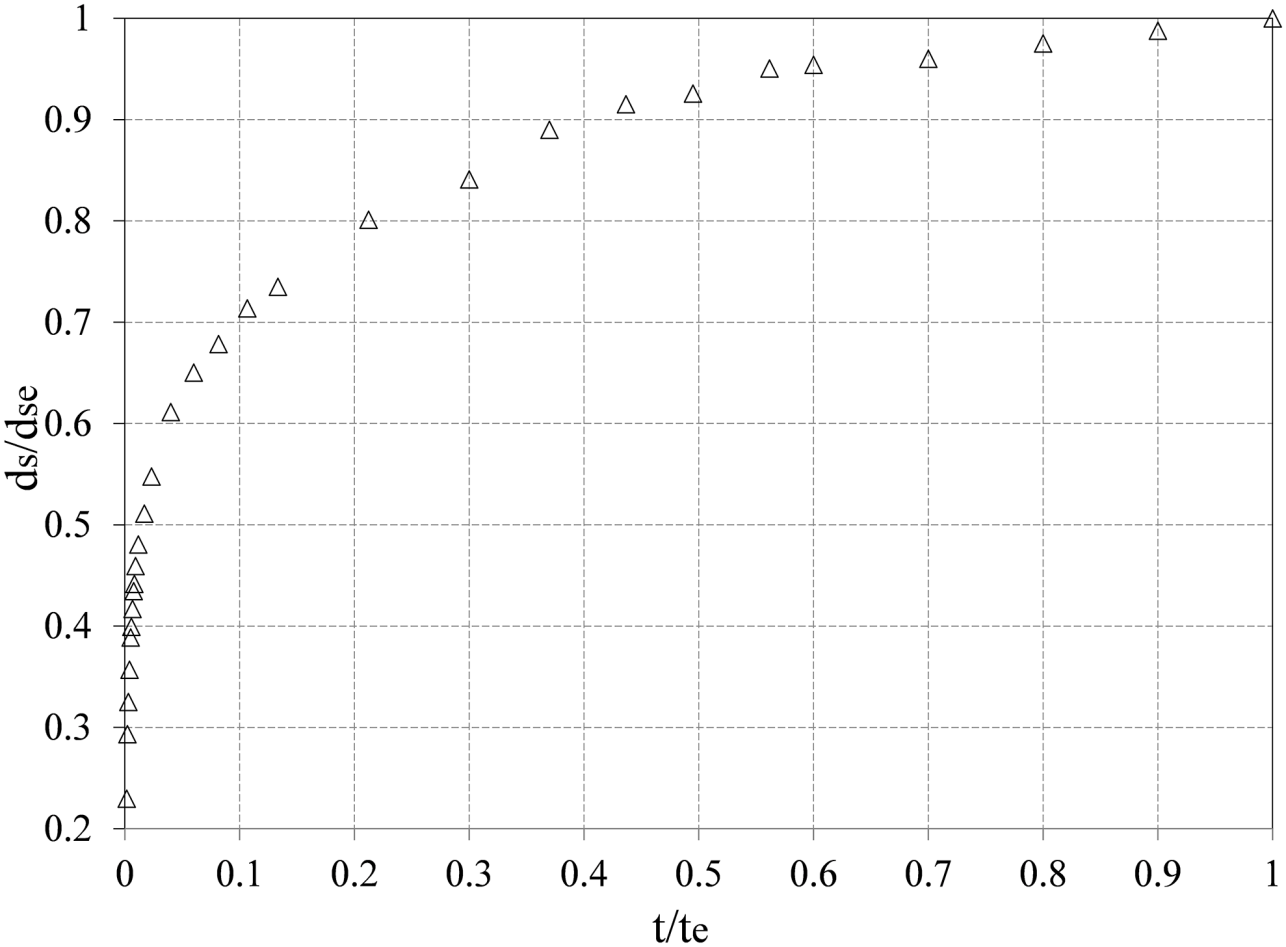
Time required for the expansion of the scour for a pier without a collar.

Figure 3 shows the required time for the scour hole to expand around the modeled pier without a collar. In order to indicate the required time for the expansion of the scour, a plot was used that consisted of the ratio of 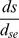 versus 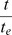 (where *d_s_* indicates the depth of the scour, *d_se_* represents the depth of scour at equilibrium state in the absence of a collar, *t* is time, and *t_e_* is the equilibrium time). Figure 3 shows that the depth of the scour varies more at the beginning of the experiment and gradually decreases until reaching the equilibrium state. Figure 3 also shows that around 80% of the scouring depth occurred during the first 20% of the equilibrium time. Under these circumstances, the scour process initially began at the upstream side of the pier and was symmetrical to the axis of the pier. The sediments was dislodged from the upstream and the lateral sides of the pier, and they were deposited at the downstream side of the pier as a heap. These heaps gradually migrated downstream. These heaps gradually migrated downstream. The duration of the experiments were set to 72 hours.

### 2. Experiments with Circular Collars

**Figure 4:**
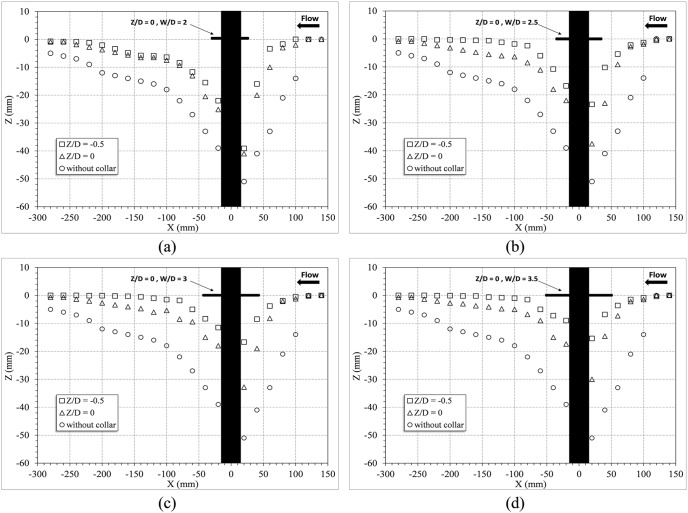
Longitudinal profiles of the bed changes around the bridge pier with the circular collars: a) W/D = 2, b) W/D = 2.5, c) W/D = 3, d) W/D = 3.5 (scale, 1∶5).

In this section, the experiments using four different sizes of circular collars are discussed. The circular collars with the dimensions of 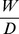 = 2.0, 2.5, 3.0, and 3.5, were installed on the pier at three different elevations, and the results were explored. Figure 4 shows the longitudinal profiles of the changes in the bed around the pier with the circular collars at the 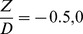 positions; in this figure, the center of the pier was considered to be X = 0. As it can be seen in Figure 4, the scour hole length was approximately 30 cm in downstream and 15 cm in upstream of the pier. Also according to the observations the width of the scour hole was 10 cm in lateral direction and it was 10 cm away from the walls of the flume. As Figure 4 shows, the collars which were placed under the streambed resulted in a greater decrease in the scour depth. Table 1 shows the percentage reduction in the depth of the scour around the bridge pier for different values of 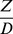 and 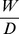. The results indicated that the percentage reduction of scour just changed 1.7% (Test 11) when the width of the circular collar exceeded 3.5D. Therefore, it is suggested that a ratio of 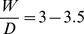 is the most effective size for circular collar in terms of scour reduction. This size range is also a more economic choice since it uses less construction materials.

**Table 1:** Characteristics of the experimental modeling, the numerical simulation and the results were obtained from each experiment.

Test No.	Collar shape	Exp/Num	Q (L/s)	U/U_cr_	D (mm)	Z (mm)	W (mm)	Z/D	W/D	A_C_/A_T_	d_se_ (mm)	R_P_ (%)
1	-	Experimental	12.2	0.95	30	-	-	-	-	-	51.0	-
2	circular	Experimental	12.2	0.95	30	15	60	0.5	2	0.75	42.7	16.3
3	circular	Experimental	12.2	0.95	30	15	75	0.5	2.5	0.84	40.9	19.8
4	circular	Experimental	12.2	0.95	30	15	90	0.5	3	0.89	38.6	24.3
5	circular	Experimental	12.2	0.95	30	15	105	0.5	3.5	0.92	37.6	26.3
6	circular	Numerical	12.2	0.95	30	15	120	0.5	4	0.94	34.6	32.2
7	circular	Experimental	12.2	0.95	30	0	60	0	2	0.75	41.0	19.6
8	circular	Experimental	12.2	0.95	30	0	75	0	2.5	0.84	37.5	26.5
9	circular	Experimental	12.2	0.95	30	0	90	0	3	0.89	32.8	35.7
10	circular	Experimental	12.2	0.95	30	0	105	0	3.5	0.92	30.0	41.2
11	circular	Numerical	12.2	0.95	30	0	120	0	4	0.94	28.1	42.9
12	circular	Experimental	12.2	0.95	30	−15	60	−0.5	2	0.75	39.1	23.3
13	circular	Experimental	12.2	0.95	30	−15	75	−0.5	2.5	0.84	23.4	54.1
14	circular	Experimental	12.2	0.95	30	−15	90	−0.5	3	0.89	16.7	67.3
15	circular	Experimental	12.2	0.95	30	−15	105	−0.5	3.5	0.92	15.4	69.8
16	circular	Numerical	12.2	0.95	30	−15	120	−0.5	4	0.94	14.2	71.5
17	Rectangular	Experimental	12.2	0.95	30	15	60	0.5	2	0.80	41.8	18.0
18	Rectangular	Experimental	12.2	0.95	30	15	75	0.5	2.5	0.87	38.1	25.3
19	Rectangular	Experimental	12.2	0.95	30	15	90	0.5	3	0.91	35.8	29.8
20	Rectangular	Experimental	12.2	0.95	30	15	105	0.5	3.5	0.94	35.1	31.2
21	Rectangular	Numerical	12.2	0.95	30	15	120	0.5	4	0.95	34.1	33.1
22	Rectangular	Experimental	12.2	0.95	30	0	60	0	2	0.80	39.3	22.9
23	Rectangular	Experimental	12.2	0.95	30	0	75	0	2.5	0.87	34.8	31.8
24	Rectangular	Experimental	12.2	0.95	30	0	90	0	3	0.91	31.4	38.4
25	Rectangular	Experimental	12.2	0.95	30	0	105	0	3.5	0.94	28.8	43.5
26	Rectangular	Numerical	12.2	0.95	30	0	120	0	4	0.95	26.6	47.8
27	Rectangular	Experimental	12.2	0.95	30	−15	60	−0.5	2	0.80	29.5	42.2
28	Rectangular	Experimental	12.2	0.95	30	−15	75	−0.5	2.5	0.87	17.9	64.9
29	Rectangular	Experimental	12.2	0.95	30	−15	90	−0.5	3	0.91	12.7	75.1
30	Rectangular	Experimental	12.2	0.95	30	−15	105	−0.5	3.5	0.94	11.5	77.5
31	Rectangular	Numerical	12.2	0.95	30	−15	120	−0.5	4	0.95	10.1	79.2

Note: D = diameter of cylindrical pier; Z = elevation of the collar in relation to streambed; W = Width and diameter of rectangular and circular collar respectively; A_C_ = net area of the collar; A_T_ = total area of collar and pier; d_se_ = equilibrium maximum scour depth; R_p_ = percentage of reduction in scour depth around the pier.

### 3. Experiments with the Rectangular Collars

**Figure 5:**
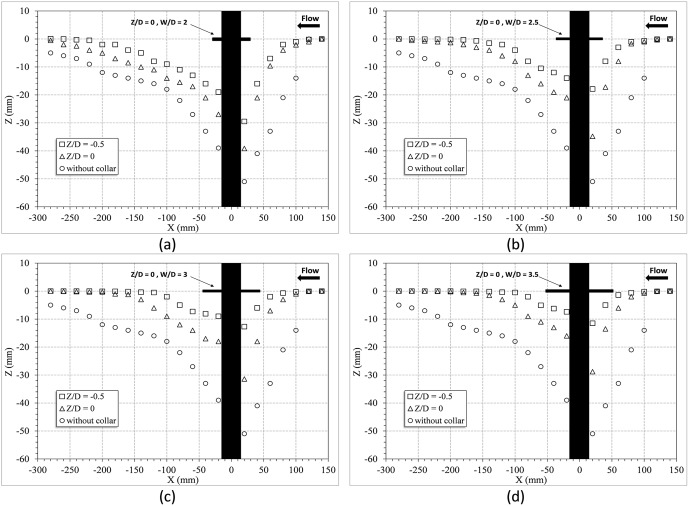
Longitudinal profiles of the bed changes around the bridge pier with the rectangular collars: a) W/D = 2, b) W/D = 2.5, c) W/D = 3, d) W/D = 3.5 (scale, 1∶5).

In this section, the experiments with four different sizes of rectangular collars are discussed. The rectangular collars had dimensions of W/D = 2.0, 2.5, 3.0, and 3.5, and they were installed on the pier at three different elevations, and the results were explored. Figure 5 shows the longitudinal profiles of the pier with rectangular collars at the 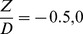 positions. The results indicated that using a collar reduced the rate of the scouring at the forepart of the pier since it controls and weakens the horseshow vortex and the rising flow. The results also showed that both the collar dimensions and the position at which the collar was mounted affect the depth of the scour. Similar to the previous section, collars with relative dimensions of 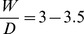 had more effective performance in terms of reducing scour depth and economy. Also, mounting the collar under the streambed decreased the scour depth more than mounting the collar above the streambed.

Experimental observations indicated that when an under-bed collar was used, first the sediments above the collar were quickly washed away, then scour around the collar took place. The depth of the scour was reduced by 78% using an under-bed collar with 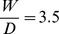.

### 4. Comparison of Rectangular and Circular Collars

**Figure 6:**
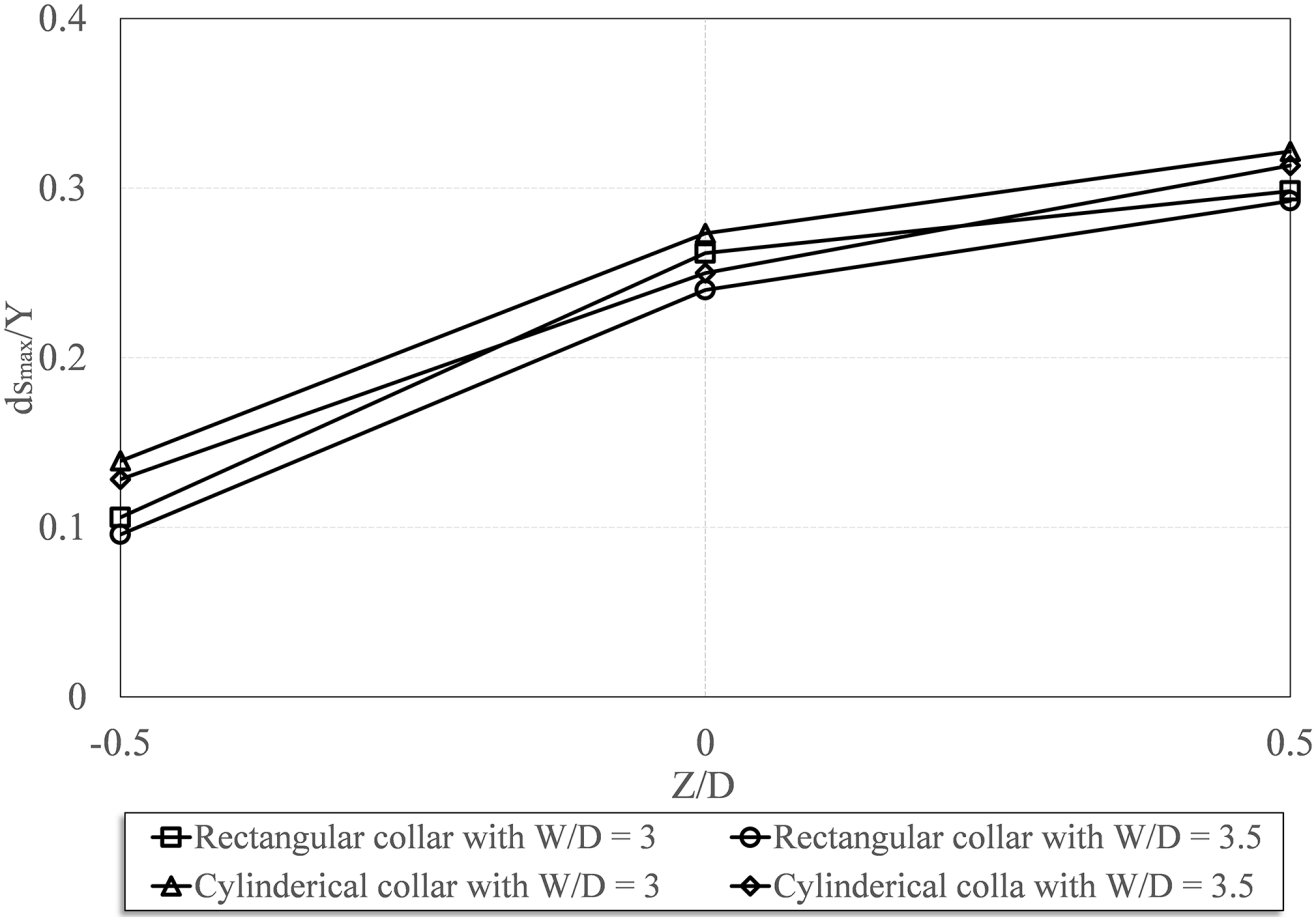
Comparison of the performances of the circular and the rectangular collars.

Figure 6 shows the comparative performances of the rectangular and circular collars in terms of reducing the scour depth around the pier. The results indicated that the rectangular collar performed better in terms of controlling and weakening the horseshow vortex and wake vortices. The rectangular collar also resulted in more reduction of the scour depth compared to the circular collar due to the existence of sharp edges inherent in its shape. The maximum decrease in the scour depth when using a rectangular collar was 79%, while it was 71% in the case when a circular collar was used (Table 1). The rectangular collars performed better than the circular collars due to the fact that the larger surface area of the rectangular collar of the same ‘diameter’ provides better coverage and protection. In addition, its corners provide larger coverage to prevent the downward flow.

### 5. Numerical Model

In this study, the SSIIM 2.0 computational fluid dynamics (CFD) model was used for three-dimensional, numerical modeling of the scour around a bridge pier. The SSIIM 2.0 model uses a finite-volume approach to discretize the equations. The water flow was computed by solving the Reynolds-averaged Navier-Stokes equations using the k-ε turbulence model. SSIIM 2.0 uses the SIMPLE method to compute the pressure [42]. It uses a power-law scheme for the discretization of the convective terms. SSIIM 2.0 computes both suspended load and bed load. The suspended sediment transport was computed by solving the transient convection-diffusion equation for sediment concentration c.

Eq. 1:
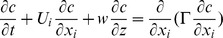



Where 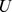 = Reynolds-averaged water velocity, 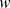 = fall velocity of the sediment, 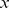 = general space dimension, 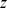 = dimension in the vertical direction, and 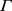 = diffusion coefficient that is set equal to the eddy viscosity taken from the 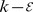 model (Olsen, 2009).

The equation describes the transport of sediment, including the effect of turbulence on reducing the settling velocity of the sediment. This equation is solved using a control-volume method on all cells, except the cell closest to the bed, where the concentration is specified by the Van Rijn formula [43]:

Eq.2:
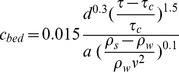



Where 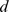 = sediment particle diameter, 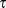 = bed shear stress, 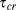 = critical bed shear stress for movement of sediment particles according to Shields’ diagram, 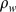 and 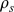 = density of water and sediment, respectively, 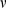 = viscosity of water and 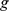 is acceleration of gravity.

In addition to the suspended load, the bed load 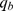 is computed by the Van Rijn (1987) formula:

Eq. 3:
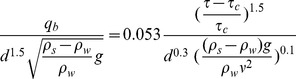



Boundary conditions were as follows: In the discharge editor, inlet and outlet water flow were defined to show the inflow and outflow discharge values. To estimate the effect of the wall on the flow, an empirical wall function, known as the standard wall function, was used (Olsen, 2009).

Eq. 4:
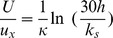



Where 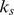 = bed roughness, 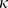 = Prandtl constant and equals to 0.4, and 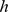 = distance from the wall [42].

### 6. Verification of the Numerical Model

**Figure 7:**
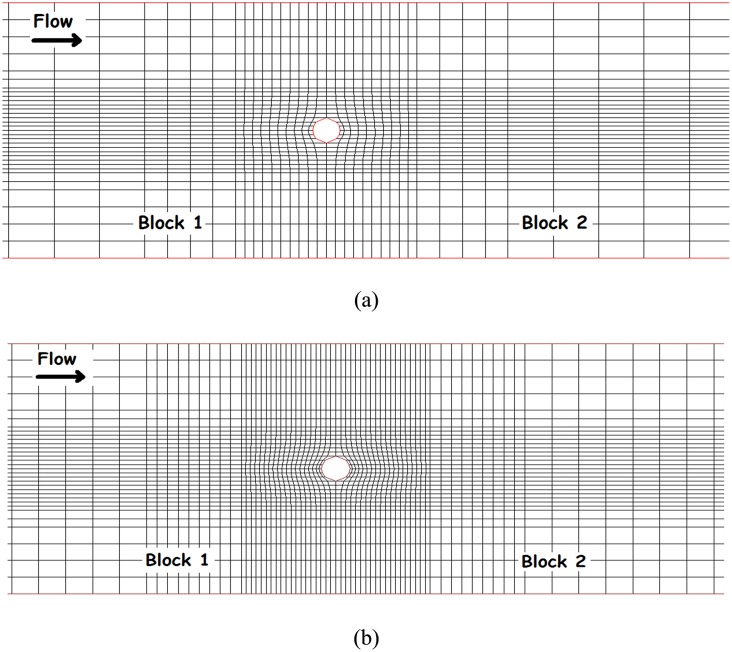
Two developed grids in SSIIM 2.0 grid editor for a single bridge pier.

In this section, four sensitivity analyses are conducted in order to investigate the effects of the various grids, bed roughness, various turbulence models, and various sediment-transport formulas. Two sizes of computational grids (82×33×11 and 136×33×11) for the experiments were used initially, and the results were compared. The distortion ratio in the finer grid around the pier was 1, while this value was changed to 2.5 in the other grid. A computer with a Core(TM) i7, 2.2 GHz processor was used to run the numerical simulations. It was found that the finer grid (136×33×11) could simulate the scour around the pier with sufficient accuracy and better than the other grid. (The coefficients of determination, R^2^, for the fine grid and the large grid for 50 points in the vicinity of the bridge pier were 0.80 and 0.68 respectively). Figure 7 shows the two grids that were developed in grid editor. Based on the sensitivity analysis on the roughness of the bed, it was found that a value of 7*d_50_* was the best value for simulating scour around a bridge pier. Various studies have indicated that the value of the roughness of the bed can range from 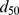 to 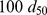 [42]. Based on the sensitivity analysis on the effect of the two turbulence models - *k-ε* standards and the *k-ε* with some RNG extensions - the latter showed the best agreement with experimental measurements. (The coefficients of determination, R^2^, for *k-ε* standards and the *k-ε* with some RNG extension were 0.58 and 0.80, respectively, in the simulations). Also, based on the sensitivity analysis on the sediment transport formulas, the results of Van Rijn’s formula had the best agreement with the measured topography. (The coefficients of determination, for Van Rijn’s formula were 0.89 and 0.84 for the experiments without a collar and with a rectangular collar, respectively).

### 7. Numerical Results

In this section, the parameters obtained in the verification section were used to simulate the effects of circular and rectangular collars on the reduction of scour around a bridge pier. In this phase, a circular and a rectangular collar with dimensions of W/D = 4 at three elevations as 
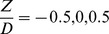
 were simulated. According to the results, increasing the width and diameter of the circular and rectangular collars to the value W = 4D provided relatively same percentage reduction of scour depth as the case for the value W = 3.5D. Table 1 shows the obtained percentage reductions for the different conditions.

### 8. Prediction of the Scour Depth Reduction Using the Collars

The main purpose of this section is to obtain an equation that can be used to predict the percentage reduction of the scour depth around a cylindrical bridge pier in presence of rectangular and circular collars. The scour depth reduction around a cylindrical bridge pier using collar depends on the geometry of the channel (channel width, channel radius and bed slope), pier dimension and pier shape, collar characteristics (collar width, collar shape, and its elevation from bed), flow conditions (flow depth and velocity), sediment properties (specific gravity, grain size, and friction angle) and fluid parameters (density and viscosity). Therefor equation 6 can be drawn for the scour depth reduction around a cylindrical bridge pier (R_p_):

Eq. 5:
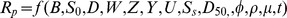
where B is width of channel, S_0_ is bed slope, D is diameter of the pier, W is width or diameter of the collar, Z is collar elevation from bed, Y is water flow depth, U is flow velocity, S_s_ is specific gravity, D_50_ is median grain size, φ is sediment fraction angle, ρ is density of fluid, µ is viscosity of fluid and t is time of the experiments.

Eq. 7 was developed by neglecting the constant parameters (*B,S_0_,Y,U,S_s_,D_50_,φ,ρ,µ,t*) and dimensional analysis of the above-mentioned parameters using the experimental and the numerical parameters.

Eq. 6:
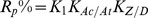



Where K_1_ is a constant value, 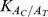 is function of Ac/A_T_, 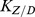 is function of Z/D, A_C_ is net area of the collar, A_T_ is total area of the collar and pier, R_p_ is percentage reduction in the scour depth around the pier.

**Figure 8:**
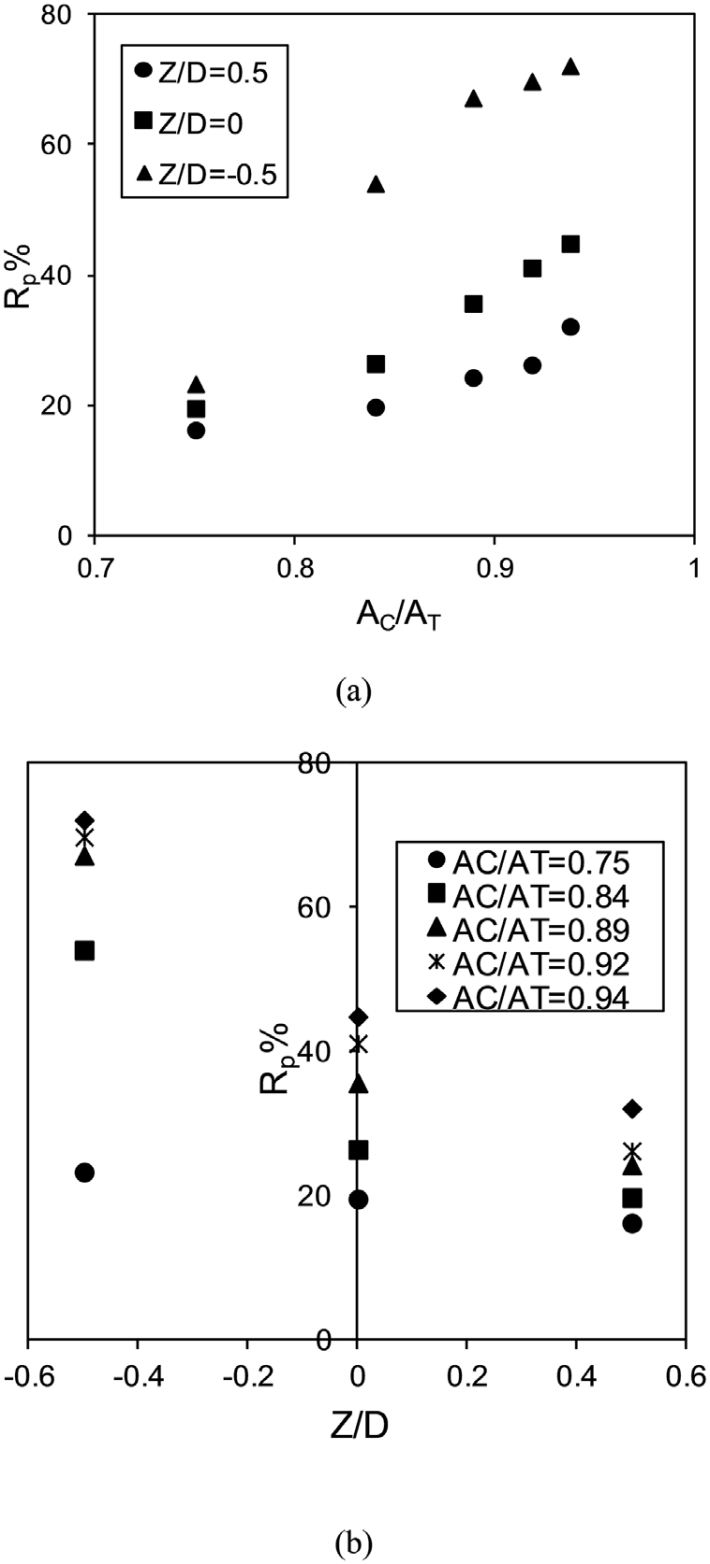
The effect of non-dimensional parameters of a circular collar on percentage reduction; a) Ac/A_T_ b) Z/D.

Figure 8 shows the effect of the non-dimensional parameters of A_c_/A_T_ and Z/D on reduction percentage of the circular collar.

Based on the effects of these parameters on reduction of the scour depth, the following functions were developed for each parameter:

Eq. 7:
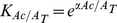



Eq. 8:
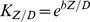
In these equations, a and b are empirical coefficients that were developed based on the experimental and the numerical data. Using the minimum square error equation (Eq 9), the values of the above coefficients were determined as follows for the cylindrical and rectangular collars:

Cylindrical: K_1_ = 0.57, a = 4.71, b  =  −0.96.

Rectangular: K_1_ = 0.90, a = 4.22, b  =  −1.00.

**Figure 9:**
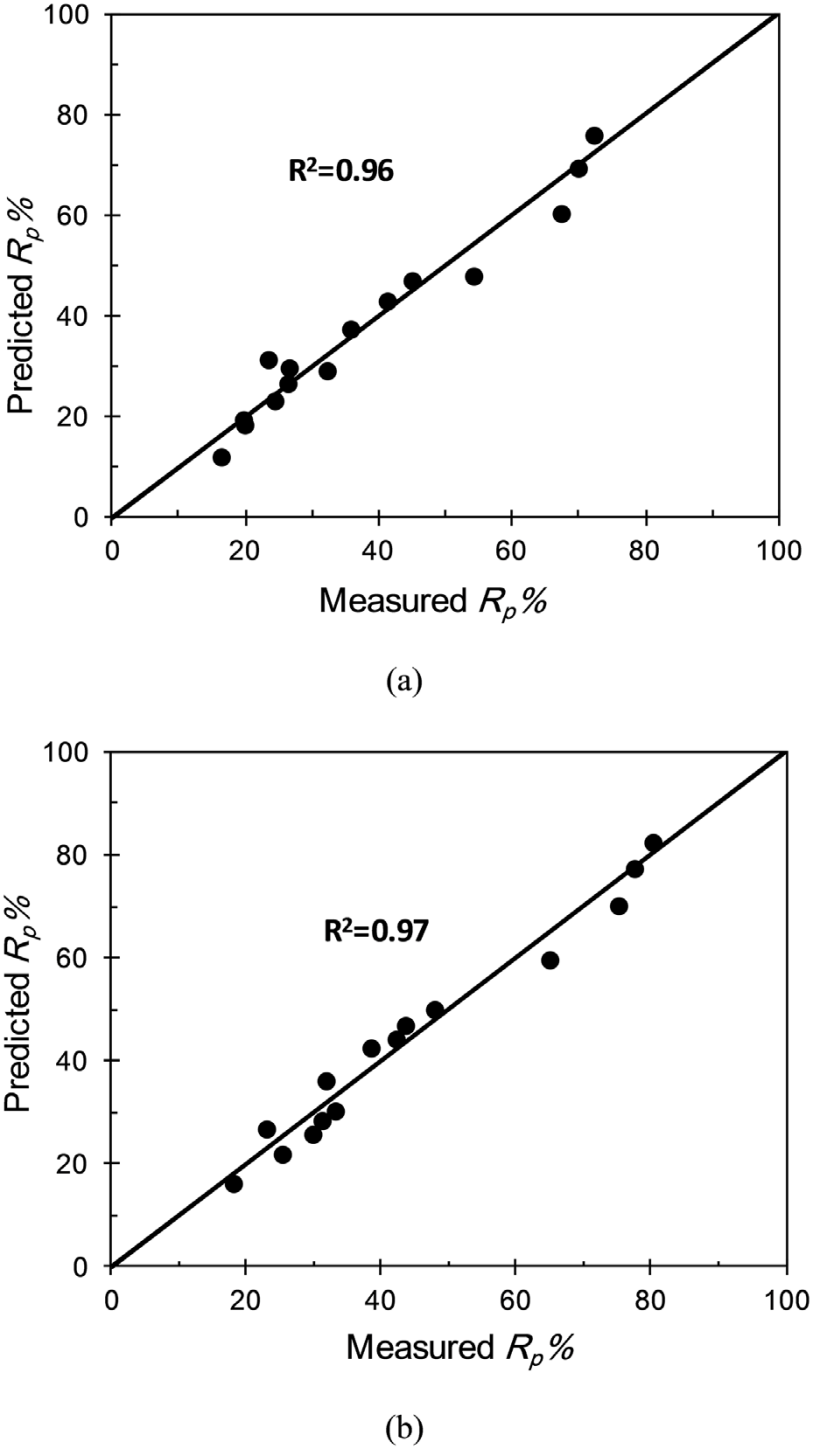
Comparison of measured and predicted values for a) a circular collar and b) a rectangular collar.

Eq. 9:
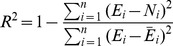



The final equations (10, 11) to predict the reduction of the scour depth around a cylindrical pier using a circular and a rectangular collar, respectively, are as follows:

Eq. 10:
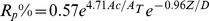



Eq. 11:
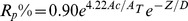



Figure 9 shows the comparison of measured and predicted values of reduction of the scour depth around the cylindrical pier for both circular and rectangular collars. It is apparent that the predicted values had an acceptable correlation with the measured values.

According to the high amount of the R^2^, these equations can be used usefully for a usual river in flood conditions including fine gravel as bed sediment.

## Conclusions

In this article, experimental and numerical methods were used to investigate the application of circular and rectangular collars with different dimensions and levels of installation relative to the streambed. This investigation was carried out in order to assess the reduction of local scour around a single, cylindrical bridge pier. The results of comparison between the performances of both types of the collars under different conditions reveals that using a collar is an effective way to reduce the depth of the scour. The following conclusions are drawn from the results of this investigation.

1. Around 80% of the scouring was observed during the first 20% of the equilibrium time. Also, the maximum rate of scouring occurred during the first hours of the experiments, and the rate of scouring decreased with time.
2. Under-bed collars reduced the scour depth more than collars that were placed on or above the stream bed.
3. The rectangular collar acts better in terms of controlling and weakening both the horseshow vortex and the rising flow, and reducing the scour depth, compared to the circular collar.
4. The best and most economical dimensions of the collars for maximum reduction of scour were found to be in the range of W = 3D to W = 3.5D.
5. The k-ε turbulence model with some RNG extensions for calculating sediment transport around a cylindrical pier had the best agreement with experimental measurements.
6. A roughness value of 7d50 showed the best agreement with the experimental results.
7. The comparison of computed and measured changes in the stream bed around a cylindrical pier indicated that the results provided by Van Rijn’s sediment transport formula had the best agreement with experimental results.
8. Based on the both acquired experimental and numerical data, two new equations were developed for predicting the effect of a collar on the percentage reduction of scour.
